# Evaluation of the Field Performance of ImmunoCard STAT!^®^ Rapid Diagnostic Test for Rotavirus in Dadaab Refugee Camp and at the Kenya–Somalia Border

**DOI:** 10.4269/ajtmh.16-0885

**Published:** 2017-06-07

**Authors:** Maurice Ope, Raymond Nyoka, Ahmed Unshur, Fredrick O. Oyier, Shafe A. Mowlid, Brian Owino, Steve B. Ochieng, Charles I. Okello, Joel M. Montgomery, Burton Wagacha, Aleksandar Galev, Abdikadir Abdow, Mathew D. Esona, Jacqueline Tate, David Fitter, Susan T. Cookson, Balajee Arunmozhi, Nina Marano

**Affiliations:** 1 U.S. Centers for Disease Control and Prevention, Nairobi, Kenya; 2 Kenya Medical Research Institute, Dadaab, Kenya; 3 United Nations High Commissioner for Refugees, Nairobi, Kenya; 4 International Organization for Migration, Nairobi, Kenya; 5 U.S Centers for Disease Control and Prevention, Atlanta, Georgia

## Abstract

Rotavirus commonly causes diarrhea in children, leading to hospitalization and even death. Rapid diagnostic tests are feasible alternatives for determining rotavirus outbreaks in refugee camps that have inadequate laboratory capacity. We evaluated the field performance of ImmunoCard STAT!^®^ Rotavirus (ICS-RV) in Dadaab Refugee Camp and at the Kenya–Somalia border. From May to December 2014, we prospectively enrolled children aged < 5 years hospitalized with acute diarrhea, defined as ≥ 3 episodes of loose stool in 24 hours for < 7 days. Stool samples were collected and tested by trained surveillance clerks using ICS-RV per manufacturer's instructions. The field performance characteristics of ICS-RV were evaluated against the gold standard test, Premier^™^ Rotaclone^®^ enzyme immunoassay. The operational characteristics were evaluated using World Health Organization (WHO) ASSURED criteria to determine whether ICS-RV is appropriate as a point-of-care test by administering a standard questionnaire and observing surveillance clerks performing the test. We enrolled 213 patients with a median age of 10 months (range = 1–48); 58.2% were male. A total of 71 (33.3%) and 60 (28.2%) patients tested positive for rotavirus infection by immunoassay and ICS-RV, respectively. The sensitivity, specificity, and positive and negative predictive values of ICS-RV compared with the immunoassay were 83.1% (95% confidence interval [CI] = 72.3–91.0), 99.3% (95% CI = 96.1–100), 98.3% (95% CI = 91.1–100), and 92.1% (95% CI = 86.6–95.5), respectively. The ICS-RV fulfilled the WHO ASSURED criteria for point-of-care testing. ICS-RV is a field-ready point-of-care test with good field performance and operational characteristics. It can be useful in determining rotavirus outbreaks in resource-limited settings.

## Introduction

Every year, 1.7 billion cases of diarrhea and 800,000 diarrhea-associated deaths occur worldwide among children aged < 5 years.^1^,^2^ In refugee camps in Africa, diarrhea remains a major cause of childhood morbidity and mortality, accounting for 10% of morbidity, 7% of deaths, and an estimated incidence of 35.5 cases/1,000 population/month in children < 5 years.^3^ Poor hygiene and sanitation and inadequate safe water supplies increase the vulnerability of children living in refugee camps to diarrheal illness.^4^–^6^

Rotavirus is an acute viral infection that is transmitted by the fecal–oral route.^7^,^8^ It is a common cause of diarrhea in children that can result in severe dehydration and necessitate hospitalization.^8^,^9^ The World Health Organization (WHO) estimates that worldwide in 2013 rotavirus caused approximately 215,000 child deaths.^10^ Aside from supportive care, including rehydration therapy, there is no specific treatment of rotavirus infection; however, several vaccines are available to prevent severe rotavirus diarrhea.^11^ In June 2009, WHO recommended the inclusion of rotavirus vaccine in national immunization programs, especially in countries where children are at high risk of severe disease, as part of the strategy to control rotavirus-associated diarrheal diseases.^12^ In July 2014, Kenya introduced the rotavirus vaccine (Rotarix^™^, GlaxoSmithKline, Rixensart, Belgium) in its national immunization schedule, which included refugee populations.^13^

Diagnosis of rotavirus infection requires laboratory confirmation, presenting a challenge in refugee camps, which often are located in remote areas with limited laboratory capabilities.^9^ Accurate and reliable point-of-care rapid diagnostic tests (RDTs) for rotavirus offer a feasible alternative for confirmation of infection and detection of rotavirus outbreaks. ImmunoCard STAT!^®^ Rotavirus (ICS-RV) is an RDT that detects rotavirus antigens in stool samples using immunogold-based technology in a horizontal-flow membrane.^14^ When initially evaluated in a laboratory setting in 1997, ICS-RV was reported to have high sensitivity (94.0%) and high specificity (100%) as well as high positive (100%) and negative (93.4%) predictive values when compared with polymerase chain reaction (PCR) tests.^14^ To date, there have been no published studies of ICS-RV performance in field settings in refugee camps.

We sought to determine the field performance characteristics of ICS-RV compared with Premier^™^ Rotaclone^®^ enzyme immunoassay (enzyme-linked immunosorbent assay [ELISA]) as the gold standard. We also sought to evaluate the operational characteristics of ICS-RV using the WHO ASSURED criteria: affordable, sensitive, specific, user-friendly, rapid and robust, equipment-free, and deliverable.^15^

## Materials and Methods

### Study sites: Dadaab Refugee Camp and Liboi Health Center at the Kenya–Somalia border.

**Figure 1.**
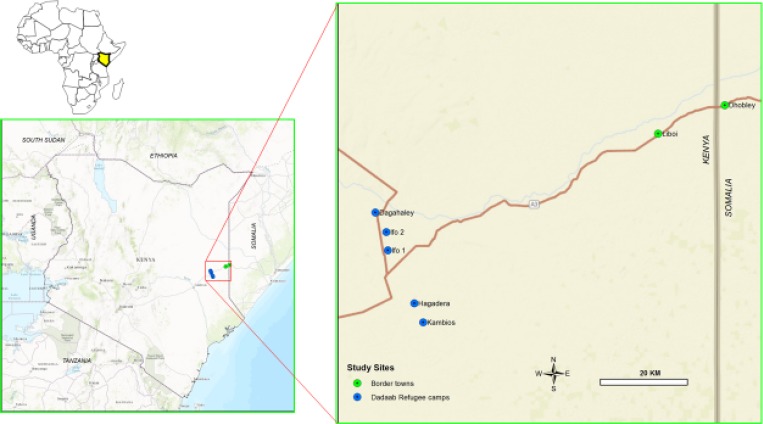
Map of Kenya showing Dadaab Refugee Camp.

Dadaab Refugee Camp is in Kenya, near the Somalia border, approximately 500 km from Kenya's capital, Nairobi. The camp hosts refugees who fled various conflicts in the greater East Africa region, including civil war in southern Somalia. As of December 2014, Dadaab Refugee Camp had an estimated population of 356,014, the majority of whom were Somali (95.2%) in origin; 16.6% were children aged < 5 years.^16^ Dadaab is a complex of five camps: Hagadera, Dagahaley, Ifo, Ifo 2, and Kambioss (Figure 1

). Each camp contains a number of outpatient health posts as well as a main hospital with inpatient and referral services.

The town of Liboi, located in Kenya approximately 80 km from Dadaab Refugee Camp and 18 km from the Kenya–Somalia border, is a transit point to more distant towns and villages in both countries. Liboi Health Center, operated by the Kenya Ministry of Health, has a 17-bed inpatient capacity. The facility serves mostly residents of Liboi and ill travelers in transit between Kenya and Somalia. Liboi's population is 17,140, consisting mainly of Somalis; 20% are children aged < 5 years. Disease outbreaks that originate within Somalia are frequently detected in frontier towns like Liboi before cases are detected at interior locations in Kenya.

### Study design.

From May 19 to December 31, 2014, trained surveillance clerks prospectively enrolled children aged < 5 years hospitalized with acute diarrhea. Acute diarrhea was defined as passage of three or more loose stools in 24 hours for < 7 days. The study sites were hospitals within Dadaab Refugee Camp and a health facility near the Kenya–Somalia border. The surveillance clerks identified eligible hospitalized participants and obtained informed consent from parents or guardians of participants. Each participant's parent or guardian was administered a standard questionnaire that sought information about the patient's demographic information and clinical signs and symptoms. Hydration status of the children was classified using the criteria described in the WHO Integrated Management of Childhood Illness guidelines for children aged 2–59 months.^17^ Whole stool samples were collected from all participants for rotavirus antigen testing. The surveillance clerks tested each specimen on site for rotavirus using the point-of-care test, ICS-RV (Meridian Bioscience Inc., Cincinnati, OH). An aliquot of the stool specimen was transported at 2–8°C to Kenya Medical Research Institute (KEMRI)/Centers for Disease Control and Prevention (CDC) laboratory in Hagadera Camp within 4 hours of collection and tested for rotavirus antigen by Premier Rotaclone ELISA (Meridian Bioscience Inc.). The operational characteristics of ICS-RV were evaluated using standardized questionnaires that sought information on how the surveillance clerks felt about the friendliness of the kit to use, time it took to perform the test, and ease of transportation and storage of the kit at point of care, using a 5-point Likert-type scale of excellent, good, average, fair, and poor. We also observed the surveillance clerks perform the point-of-care test to identify any difficulties in handling the reagents and measurements required for the test.

### Rapid diagnostic testing.

All samples were tested in the field by trained surveillance clerks using ICS-RV per manufacturer's instructions. Briefly, the reagents were brought to room temperature (20–30°C). Then 25 μL of stool sample was diluted with 350 μL of sample diluent provided by the manufacturer. The sample was mixed well by shaking by hand for 10 seconds. The ICS-RV device was labeled with the patient identification code, and 150 μL of diluted sample was drawn and added to the sample port in the device. The specimen was incubated at an uncontrolled room temperature for 10 minutes, during which time the diluted specimen moved past the control zone. The temperature in the study area during the study period was estimated to have ranged from a minimum of 21°C to a maximum of 34.8°C.^18^ The control and test zones were visually read for the presence or absence of a red/purple line at the end of 10 minutes. A sample was considered to have rotavirus antigens and reported as positive if there was a visually detectable red/purple line in both the test and control zones, whereas a sample was reported as negative if there was a detectable red/purple line only in the control zone but not in the test zone. If a sample returned an invalid result (no visually detectable red/purple line in the control zone), the test procedure was repeated using a new device and a rediluted specimen from the original stool sample.

### Premier Rotaclone enzyme immunoassay.

To ensure optimal use of resources, stool samples were stored at −25°C and tested for rotavirus in batches of a minimum of 20 samples using a commercial enzyme immunoassay (Premier Rotaclone ELISA) following the manufacturer's instructions. This assay uses murine monoclonal antibody directed against rotavirus antigen. A positive and a negative control were included in all runs. Results were read spectrophotometrically at 450 nm. Samples with an optical density value of > 0.150 were reported as positive as recommended by the manufacturer in the package insert. The run was repeated if the controls did not fall within the expected range. It is noteworthy that the laboratorian who performed the Premier Rotaclone ELISA test was not blinded to the ICS-RV result.

### Ethical approval.

The protocol was approved by the institutional review boards (IRBs) of the KEMRI (Scientific Steering Committee no. 2715) and the U.S. CDC (Protocol no. 6687): CDC's human subjects research office relied on KEMRI for IRB oversight.

### Data management.

Data were entered into a Microsoft Access database (Microsoft Corp., Redmond, WA). Epidemiological data and laboratory results were linked using unique identifiers. Data were analyzed using SAS (SAS version 9.3; Cary, NC), including calculation of sensitivity, specificity, and positive value and negative predictive values, with 95% confidence intervals (CI).

## Results

### Demographics and clinical characteristics.

We enrolled 213 patients who met the study case definition. We collected stool samples from all participants and tested the samples for rotavirus by both ICS-RV and Premier Rotaclone ELISA. The median age of patients with suspected rotavirus was 10 months; 124 (58.2%) were male. Among patients with suspected rotavirus, 104 (48.8%) had dehydration (Table 1). None of the enrolled participants died during hospitalization. There was no significant difference in characteristics between patients confirmed as rotavirus-infected and noninfected by the ELISA test.

### Field performance characteristics of ICS-RV.

By Premier Rotaclone ELISA, a total of 71 (33.3%) participants had confirmed rotavirus infection and 142 (66.7%) had a negative test result. By ICS-RV, 60 (28.2%) patients tested positive and 152 (71.3%) had a negative test result. Of all tested, one (0.5%) specimen tested by ICS-RV had an invalid result; however, a repeat test was not done as required; this specimen tested negative with Premier Rotaclone ELISA. ICS-RV returned a false-positive result in 1/141 (0.7%) patients and a false-negative result in 12/71 (16.9%) patients (Table 2). Excluding the single invalid test, the sensitivity of ICS-RV was 83.1% (95% CI = 72.3–91.0), specificity 99.3% (95% CI = 96.1–100), positive predictive value 98.3% (95% CI = 91.1–100), and negative predictive value 92.1% (95% CI = 86.6–95.5). The sensitivity, specificity, and positive and negative predictive values of the test did not change significantly when severity of dehydration was taken into account (Table 2).

### Operational characteristics of ICS-RV.

The study coordinator observed while 16 surveillance clerks performed the ICS-RV test. The median number of tests that the surveillance clerks had performed before assessment by the coordinator was 6 (range = 1–28). The surveillance clerks took a median of 10 minutes (range = 8–15) to perform the test. The time taken by each surveillance clerk to complete testing of specimens decreased with each test performed. The surveillance clerks felt that they could perform as many as 30–50 tests in an 8-hour working day. All surveillance clerks perceived the ICS-RV as user-friendly and rated the ease of opening the kit and reading results as excellent. Half the surveillance clerks (8/16) rated the ease of handling the reagents used for performing the test as excellent, whereas the other half rated it as good. The average time needed to train a surveillance clerk to perform the test using 12 simplified testing steps was 40 minutes. The test kit did not require specialized equipment to perform; the only requirement was storage in a cool box at a temperature of 2–8°C. The estimated unit cost of performing the test was US$9.70, including shipment and RDT costs but not personnel time. The majority of the surveillance clerks (12/16) also rated the ease of delivery of the test kit to the field sites as excellent, whereas the remaining four of 16 rated it as “good.”

## Discussion

Our study demonstrated that, in a refugee camp setting, the ICS-RV fulfilled the WHO ASSURED criteria for point-of-care testing: affordable, sensitive, specific, user-friendly, rapid and robust, equipment-free, and deliverable. The ICS-RV was affordable, costing less than US$10, compared with the gold standard test, which is estimated to cost US$19.10 per test and in addition requires an ELISA machine and a highly skilled laboratory technician to perform. We found that ICS-RV had good sensitivity (83.1%, 95% CI = 72.3–91.0) and excellent specificity (99.3%, 95% CI = 96.1–100). Moreover, only one of 141 specimens (0.7%) yielded a false-positive result. Our study also demonstrated that ICS-RV was user-friendly; surveillance clerks did not need extensive training to perform the test accurately and were able to perform the test using simplified instructions. The training was hands-on, lasted approximately 40 minutes, and included both demonstration and observing participants perform the test. Results were generated within 10 minutes, thus providing clinically important feedback to healthcare providers while the patient was still at the clinic. Other than test tubes and a timer, no special equipment was required to perform the test. The point-of-care test could be delivered to remote settings using only a cool box at a temperature of 2–8°C. ICS-RV shows promise in overcoming practical challenges such as the need to batch samples, the long turnaround time, and the high costs associated with rotavirus testing using the gold standard ELISA methods.^14^

Unlike laboratory-based studies, where there is greater control of variables, field-based evaluations are performed in real-world conditions and therefore can more realistically demonstrate the practical value of the RDT. This study found a higher proportion of false-negative results (16.9% versus 6.0%) than was found by Dennehy and others during initial laboratory studies done in 1997.^14^ According to the manufacturer of ICS-RV, the RDT detects rotavirus antigen in stool and has a limit of detecting 1.8 × 10^6^–3.7 × 10^6^ rotavirus particles per test volume. Since infected persons are known to shed large quantities of the virus in stool before and several days after onset of symptoms,^19^ and given that we enrolled only hospitalized children with acute diarrhea, it is unclear why a higher proportion of false-negative results were found in this study.

The etiological diagnosis of rotavirus diarrhea requires laboratory confirmation. However, in settings where laboratory tests for rotavirus are not easily accessible, clinicians often rely on a combination of clinical judgment and more time-consuming laboratory tests to rule out bacterial causes of diarrhea to diagnose rotavirus infection.^11^ The mainstay of management of rotavirus-associated diarrhea is rapid rehydration with intravenous fluids or oral rehydration solutions.^11^,^20^ Overuse of antibiotics for the treatment of diarrhea in resource-limited settings such as refugee camps is common.^21^ Early diagnosis of rotavirus infection with an effective bedside RDT may potentially save valuable antibiotics, and prevent further development of antibiotic-resistant organisms. Early diagnosis and appropriate management of rotavirus infection is important especially in humanitarian crisis settings, where pediatric patients are further compromised by poor nutrition and undervaccination for routine childhood illnesses. Early detection of rotavirus outbreaks can also facilitate rapid implementation of prevention and control measures and potentially reduce the duration of outbreaks.^22^

The primary limitation to our study was that the detection of rotavirus in stool by ICS-RV did not rule out the presence of other pathogens, and coinfections are common with diarrheal illnesses in children.^23^,^24^ It may be imprecise to compare our findings with the initial laboratory-based evaluation of ICS-RV performed in 1997, in which PCR was the gold standard, unlike our study where ICS-RV was compared with ELISA. It is notable that ELISA is considered the gold standard for rotavirus diagnosis; however, subsequent studies have demonstrated that even when diarrhea is not associated with rotavirus infection, reverse transcription PCR is able to detect low levels of rotavirus in ELISA-negative stools.^25^,^26^

As Dadaab lacks capacity for rotavirus ELISA because trained laboratory staff are few and supplies and equipment for ELISA testing are costly, the RDT would be an excellent diagnostic tool to improve diagnosis of rotavirus-associated diarrhea hence optimal use of resources in such settings. Furthermore, it could be used in the evaluation and monitoring of vaccine effectiveness in such settings. A reduction in the RDT's cost can remarkably improve access and thus enhance its usefulness. Although there is need to develop a combination RDT that can test for multiple pathogens (both bacterial and viral), this RDT is a field-ready, point-of-care diagnostic with good utility in resource-limited and remote settings.

## Figures and Tables

**Table 1:** Comparison of demographic and clinical characteristics of enrolled participants, Dadaab Refugee Camp and Kenya–Somalia border, 2014

	Suspected rotavirus (*N* = 213)	Rotavirus ELISA positive (*N* = 71)	Rotavirus ELISA negative (*N* = 142)	*P* value
Median age (range)	10 (1–48) months	10 (3–48)	10 (2–48)	0.66
Male	124 (58.2%)	40 (56%)	84 (60%)	0.69
Dehydration status				0.16
Severe dehydration	49 (23.0%)	20 (28.2%)	29 (20.4%)	
Some dehydration	55 (25.8%)	13 (18.3%)	42 (29.6%)	
No dehydration	109 (51.2%)	38 (53.5%)	71 (50.0%)	

ELISA = enzyme-linked immunosorbent assay.

**Table 2:** Field performance characteristics of ImmunoCard STAT!^®^ Rotavirus compared with Premier^™^ Rotaclone^®^ enzyme immunoassay for the detection of rotavirus, Dadaab Refugee Camp, Kenya, 2014

	True positive	True negative	False positive	False negative	Sensitivity % (95% CI)	Specificity % (95% CI)	Positive predictive value % (95% CI)	Negative predictive value % (95% CI)
Severe dehydration	17	28	0	3	85.0 (62.1−96.8)	100.0 (87.7–100)	100.0 (80.5–100)	90.3 (74.2–98.0)
Some dehydration	9	41	1	4	69.2 (38.6−90.9)	97.6 (87.4–99.9)	90.0 (55.5–99.7)	91.1 (78.8–97.5)
No dehydration	33	71	0	5	86.8 (71.9−95.6)	100 (94.9–100)	100 (89.4–100)	93.4 (85.3–97.8)
Overall	59	140	1	12	83.1 (72.3−91.0)	99.3 (96.1–100)	98.3 (91.1–100)	92.1 (86.6–95.5)

CI = confidence interval.
